# Evaluation of the Ameliorative Potential of 3,5-*bis*(2-hydroxyethyl)-1,3,5-thiadiazinane-2-thione against Scopolamine-Induced Alzheimer’s Disease

**DOI:** 10.3390/ijms25169104

**Published:** 2024-08-22

**Authors:** Shagufta, Gowhar Ali, Adnan Khan, Abdur Rasheed, Farah Deeba, Rahim Ullah, Muhammad Shahid, Haleema Ali, Rasool Khan, Najeebullah Shamezai, Naveed Sharif

**Affiliations:** 1 Department of Pharmacy, University of Peshawar, Peshawar 25120, Pakistan; phr120102041@gmail.com (S.); rphabdur.rasheed@uop.edu.pk (A.R.); fari.khan931@gmail.com (F.D.); najeebpharmacist@gmail.com (N.S.); 2 DHQ Teaching Hospital Timergara, Lower Dir, Khyber Pakhtunkhwa (KPK), Timergara 18300, Pakistan; 3 Department of Pharmacy, Sarhad University of Science and Information Technology, Peshawar 25000, Pakistan; yousafzai.rahimullah@yahoo.com; 4 Department of Pharmacy, CECOS University of IT and Emerging Sciences, Peshawar 25000, Pakistan; shahidsalim_2002@hotmail.com; 5 Institute of Chemical Sciences, University of Peshawar, Peshawar 25120, Pakistan; haleema.chemist@gmail.com (H.A.); rasoolkhan1@hotmail.com (R.K.); 6 Institute of Pathology and Diagnostic Medicine, Khyber Medical University Peshawar, Khyber Pakhtunkhwa (KPK), Peshawar 25100, Pakistan; changulpk@gmail.com

**Keywords:** Alzheimer’s disease, THTT, scopolamine, acetyl choline esterase

## Abstract

Alzheimer’s disease (AD) is the most common neurodegenerative disorder, marked by cognitive impairment. Currently, the available treatment provides only symptomatic relief and there is a great need to design and formulate new drugs to stabilize AD. In the search for a new anti-Alzheimer’s drug, 3,5-*bis*(2-hydroxyethyl)-1,3,5-thiadiazinane-2-thione (THTT), a tetrahydro-2H-1,3,5-thiadiazine-2-thione derivative, was investigated against a scopolamine-induced Alzheimer’s model. The selected test compound was administered intraperitoneally in three doses (15 mg/kg, 30 mg/kg, and 45 mg/kg). The test compound exhibited an IC50 value of 69.41 µg/mL, indicating its ability to inhibit the acetylcholinesterase enzyme. An antioxidant DPPH assay revealed that the IC50 value of the test compound was 97.75 µg/mL, which shows that the test compound possesses antioxidant activity. The results of behavior tests including the Y-maze and elevated plus maze (EPM) show that the test compound improved short-term memory and spatial memory, respectively. Furthermore, in the Morris water maze (MWM) and light/dark model, the test compound shows improvements in learning and memory. Moreover, the results of histological studies show that the test compound can protect the brain against the harmful effects of scopolamine. Overall, the findings of our investigation suggest that our chosen test compound has disease-modifying and neuroprotective activities against the scopolamine-induced Alzheimer’s model. The test compound may be beneficial, subject to further elaborate investigation for anti-amyloid disease-modifying properties in AD.

## 1. Introduction

Alzheimer’s disease (AD) is a neurodegenerative disorder marked by memory loss, behavioral changes, and cognitive dysfunction [1,2]. Globally, there are 33.9 million people affected by Alzheimer’s disease. The prevalence of Alzheimer’s disease rises with age, and it is projected to impact 1 out of every 85 individuals globally by the year 2050 [3]. In AD, there is a continuous neuronal deterioration in the amygdala and hippocampus, resulting in an irremediable decrease in cognitive abilities and memory [4]. The primary pathophysiological factors contributing to neuronal damage in AD include aggregation of abnormal beta-amyloid proteins, tau protein phosphorylation, and insufficient production of acetylcholine (Ach) [5].

One of the major histopathological hallmarks of AD is the extracellular accumulation and an increase in an amyloid peptide, formed by various enzymatic cleavages of the amyloid precursor protein (APP), followed by the formation of intracellular neurofibrillary tangles (NFTs) in the brain [6]. Therapeutic approaches for the management of AD are currently in practice, including rivastigmine, galantamine, donepezil, and NMDA receptor antagonists like memantine. However, enhanced research efforts are essential for effectively addressing Alzheimer’s disease, which must aim to improve therapeutic approaches and develop more advanced alternatives that can aid in preventing dementia and slowing the progression of the disease.

Our chosen test compound, 3,5-*bis*(2-hydroxyethyl)-1,3,5-thiadiazinane-2-thione (THTT), belongs to the thiadiazine class—a six-membered heterocyclic molecule comprising one sulfur and two nitrogen atoms linked to three carbon atoms. Thiadiazines have been recognized for their diverse properties, including anti-inflammatory, analgesic, and antioxidant effects. Some derivatives have shown promising potential in medical applications, serving as anti-anxiety agents, sedatives, myorelaxants, and spasmolytics [7]. Tetrahydro-2H-1,3,5-thiadiazine-2-thione, a derivative of thiadiazine, holds notable significance due to its diverse pharmacological properties. This compound and its derivatives exhibit anti-tuberculosis, anti-protozoal, anti-fungal, antibacterial, anthelmintic, and anti-cancer activities [8,9,10]. The current study was designed to evaluate the anti-Alzheimer’s potential of the test compound.

## 2. Results

### 2.1. Acute Toxicity Test

Before the in vivo studies, THTT (test drug) was tested for acute toxicity at different doses ranging from 15 mg/kg to 500 mg/kg and the animals were observed for any abnormal behavior and mortality. THTT was found to be safe at the tested doses and no acute toxicity was observed in different groups of animals at the mentioned doses; thus, the LD50 was estimated to be higher than 500 mg/kg.

### 2.2. Acetylcholinesterase Inhibition by 3,5-bis(2-hydroxyethyl)-1,3,5-thiadiazinane-2-thione

**Figure 1:**
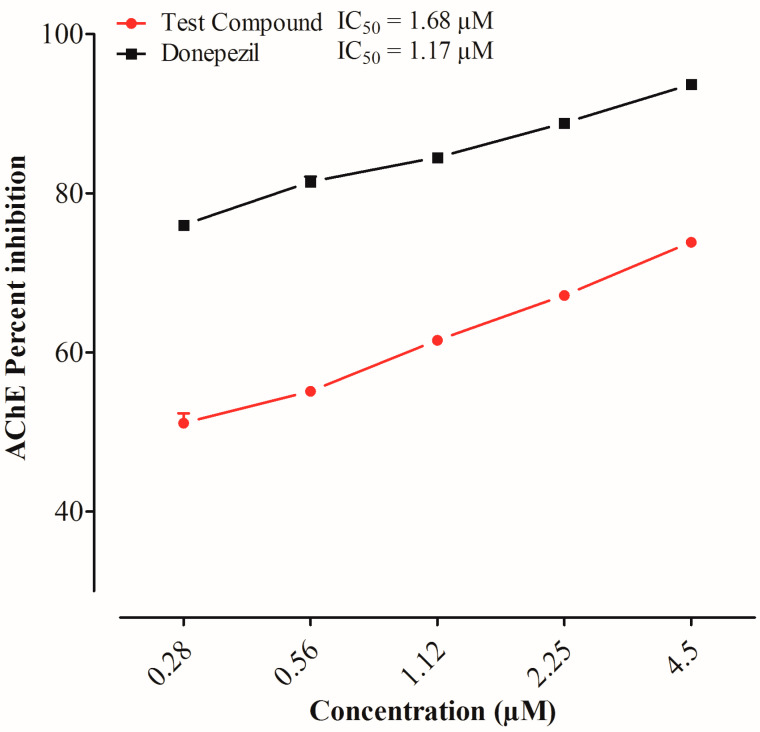
Acetylcholinesterase enzyme inhibition assay. The graph shows the results of the acetylcholinesterase enzyme inhibition assay for both the test compound and donepezil experimental groups. No significant differences between these two groups were found for any of the concentrations tested.

The test compound and donepezil showed a rise in the % inhibition of acetylcholinesterase enzyme in a concentration-dependent manner. The % inhibition of the test compound and donepezil at the highest concentration were 73.84% and 94.55%, respectively. The test compound and donepezil showed inhibition against the AChE enzyme with IC50 values of 69.41 μg/mL and 8.65 μg/mL, respectively, in a concentration-dependent manner, as shown in Figure 1.

### 2.3. 1,1-Diphenyl, 2-Picrylhydrazal Free Radical Scavenging Assay

The standard and the test drug exhibited an increase in the inhibition of the free radicals in a concentration-dependent manner. The % inhibition of free radicals by the test compound at the highest concentration was 75% with an IC50 value of 97.75 μg/mL. The percent inhibition of free radicals by ascorbic acid at the highest concentration was 80% with an IC50 value of 67.98 μg/mL, as shown in Table 1.

### 2.4. Results of the Behavioral Studies

#### 2.4.1. Effect of the Test Compound in the Elevated Plus Maze

**Figure 2:**
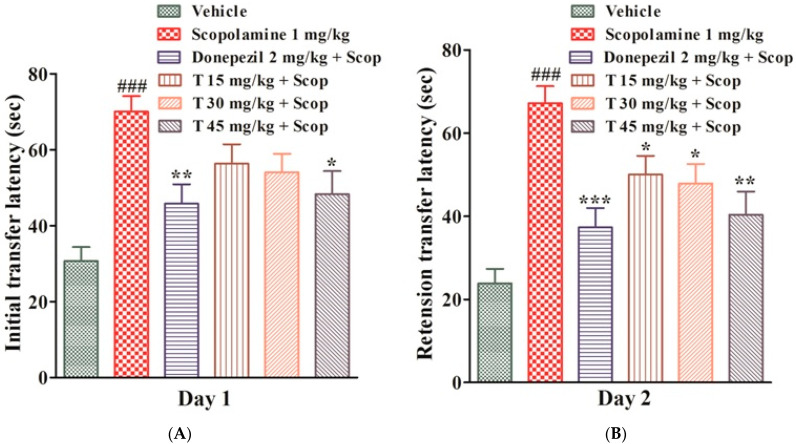
The effect of the test drug on ITL (**A**) and RTL (**B**) in EPM. All values are expressed as mean ± SEM. ^###^ *p* < 0.001 as compared to the vehicle group, * *p* < 0.05, ** *p* < 0.01, *** *p* < 0.001 as compared to the scopolamine-only group, one-way ANOVA followed by Dunnett’s post hoc test (*n* = 6).

The first-day transfer latency showed animal learning, whereas the second-day transfer latency showed memory, i.e., the retention of information. The experimental results showed that the initial transfer latency (ITL) and retention transfer latency (RTL) of the scopolamine-treated group were significantly higher (*p* < 0.001) than those of the vehicle group, which reflected memory loss. The ITL of the donepezil group (*p* < 0.01) and test compound group at a dose of 45 mg/kg (*p* < 0.05) were significantly lower than those of the scopolamine-treated group. Similarly, the RTL of the donepezil group (*p* < 0.001) and test compound group at a dose of 45 mg/kg (*p* < 0.01) were significantly less than that of the scopolamine-treated group. The test compound showed an improvement in memory in a dose-dependent manner, as shown in Figure 2.

#### 2.4.2. Effect of the Test Compound in the Y-Maze Test

**Figure 3:**
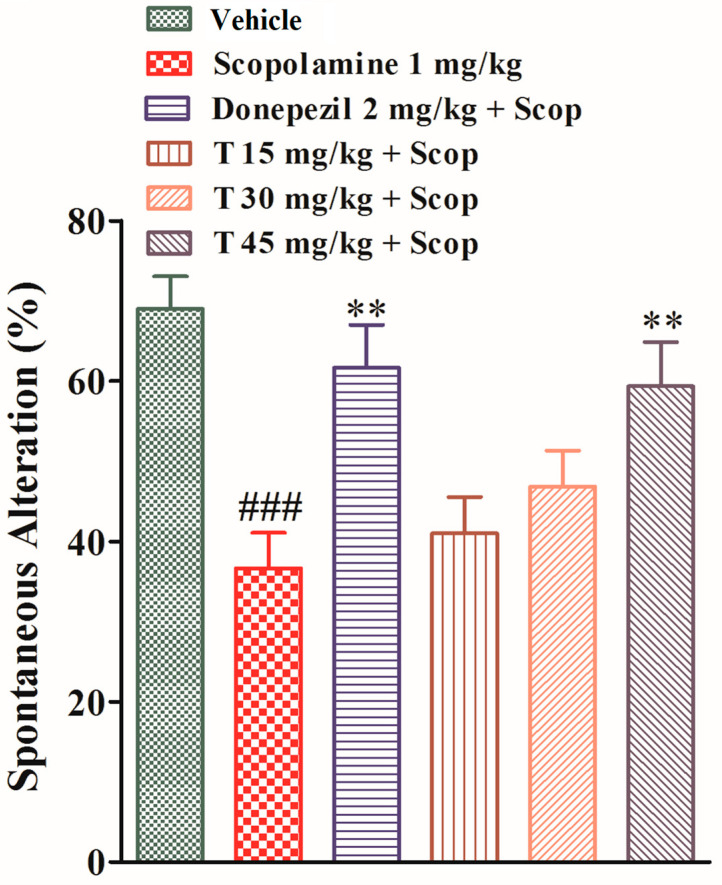
The effect of the test drug on spontaneous alteration behavior in the Y-maze. All values are expressed as mean ± SEM. ^###^ *p* < 0.001 as compared to the vehicle group, ** *p* < 0.01 as compared to the scopolamine-only group, one-way ANOVA followed by Dunnett’s post hoc test (*n* = 6).

The effects of the test compound and donepezil on the spontaneous alteration in the Y-maze test, which reflects short-term memory, were observed. The spontaneous alteration of the scopolamine-treated group was significantly (*p* < 0.001) less than that of the vehicle group, which shows the impairment of memory. The spontaneous alteration of the test compound groups at various doses (15, 30, and 45 mg/kg) and that of donepezil group were less than that of the scopolamine-treated group (*p* > 0.05, *p* < 0.01), as shown in Figure 3.

#### 2.4.3. Effect of the Test Compound in the Light and Dark Test

**Figure 4:**
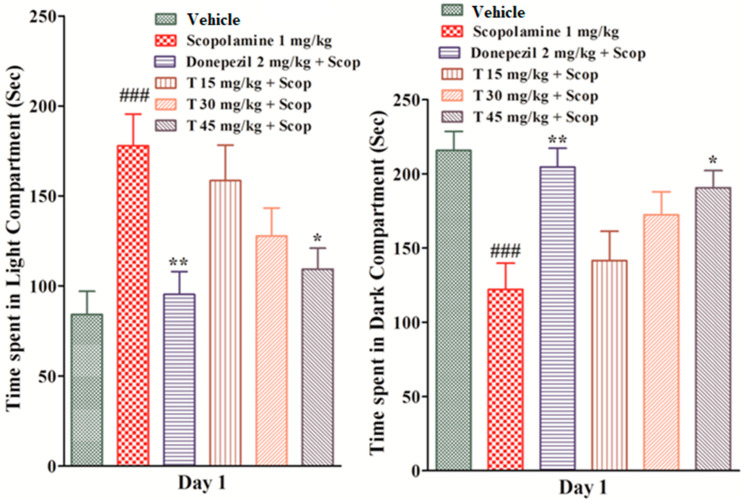
The effect of the test compound on the time spent in the light and dark compartments in light and dark experiments. All values are expressed as mean ± SEM. ^###^ *p* < 0.001 as compared to the vehicle group, * *p* < 0.05 and ** *p* < 0.01 as compared to the scopolamine-only group, one-way ANOVA followed by Dunnett’s post hoc test (*n* = 6).

The effect of the test drug on the light and dark compartments was observed. On the first day, the scopolamine-treated group spent significantly more time (*p* < 0.001) in the light compartment and significantly less time (*p* < 0.001) in the dark compartment as compared to the vehicle group. The test drug groups at doses of 15 mg/kg and 30 mg/kg spent less time in the light and more time in the dark area as compared to the scopolamine-treated group, but the values were not significant. The donepezil group (*p* < 0.01) and the test compound group at a dose of 45 mg/kg (*p* < 0.05) spent significantly less time in the light area and significantly more time in the dark area as compared to the scopolamine-treated group. On the second day, similarly, the scopolamine-treated group spent significantly more time (*p* < 0.001) in the light compartment and significantly less time (*p* < 0.0010) in the dark compartment as compared to the vehicle group, which shows an impairment of memory (Figure 4).

#### 2.4.4. Effect of the Test Compound in the MWM Test

**Figure 5:**
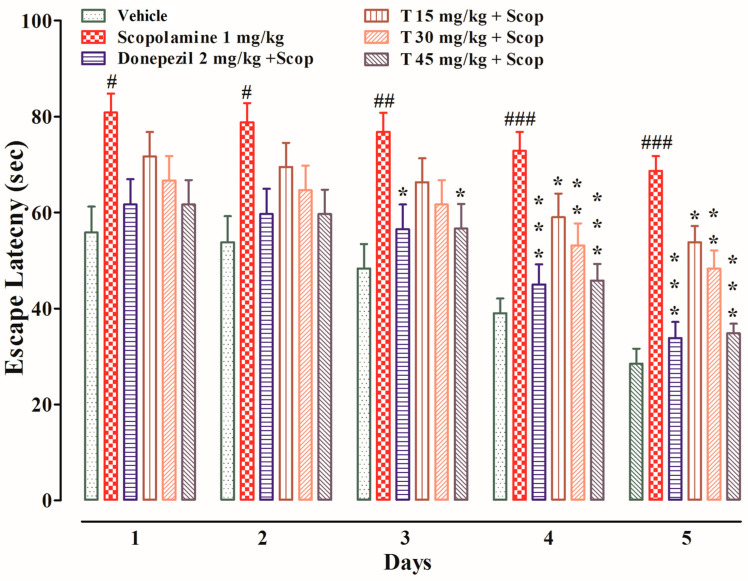
The effect of the test compound on the escape latency in the Morris water maze. All values are expressed as mean ± SEM. The “#” and “##” symbols indicate significance levels in the scopolamine-treated group: “#” denotes *p* < 0.05, “##” denotes *p* < 0.01, and “###” denotes *p* < 0.001. ^###^ *p* < 0.001 as compared to the vehicle group, * *p* < 0.05, ** *p* < 0.01, and *** *p* < 0.001 as compared to the scopolamine-only group, one-way ANOVA followed by Dunnett’s post hoc test (*n* = 6).

**Figure 6:**
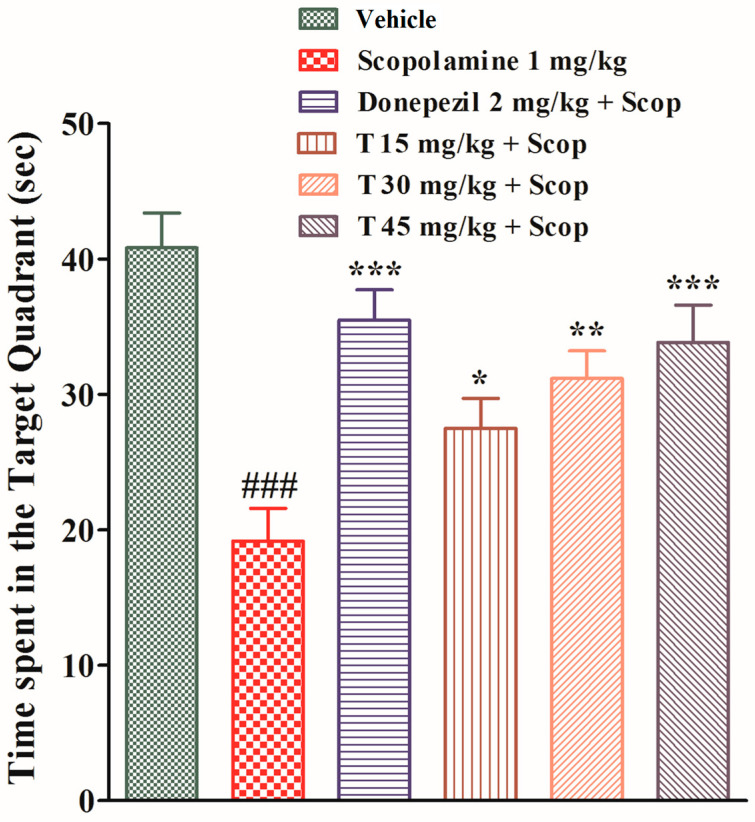
The effect of the test compound on the time spent in the target quadrant in the Morris water maze. All values are expressed as mean ± SEM. ^###^ *p* < 0.001 as compared to the vehicle group, * *p* < 0.05, ** *p* < 0.01, and *** *p* < 0.001 as compared to the scopolamine-only group, one-way ANOVA followed by Dunnett’s post hoc test (*n* = 6).

The effects of the test compound on the escape latency and the time spent in the target quadrant are shown in Figure 5 and Figure 6, respectively. During the test trial, i.e., from days one to five, the scopolamine-treated group took significantly longer (*p* < 0.05 on the first and second days, *p* < 0.01 on the third day, and *p* < 0.001 on the fourth and fifth days) to find the platform as compared to the vehicle group. The donepezil-treated group took significantly less time (*p* < 0.05 on the third day and *p* < 0.001 on the fourth and fifth days) to find the platform as compared to the scopolamine-treated group. Similarly, the test compound group at a dose of 15 mg/kg took significantly less time (*p* < 0.05 on the fourth and fifth days) to find the platform in comparison to the scopolamine-treated group. Likewise, the test compound group at a dose of 30 mg/kg took significantly less time (*p* < 0.01 on the fourth and fifth days) to find the platform in comparison to the scopolamine-treated group. In keeping with this, the test compound group at a dose of 45 mg/kg took significantly less time (*p* < 0.05 on the third day and *p* < 0.001 on the fourth and fifth days) to find the platform as compared to the scopolamine-treated group. During the probe test, the scopolamine-treated group spent significantly less time in the target quadrant as compared to the vehicle group. The test compound groups at doses of 15 mg/kg (*p* < 0.05) and 30 mg/kg (*p* < 0.01) spent significantly more time in the target quadrant as compared to the scopolamine-treated group. Similarly, both the test compound group at 45 mg/kg and the donepezil-treated group spent significantly (*p* < 0.001) more time in the target quadrant as compared to the scopolamine-treated group.

#### 2.4.5. Effect of the Test Compound in the Open Field Test

In an open field experiment, all experimental animals from different groups were exposed to the open arena of the apparatus, to determine the effect of the test drug on the locomotor activity, and each rearing and line crossing was recorded. The scopolamine-treated group crossed a significantly greater number of lines (*p* < 0.001) and showed significantly less rearing (*p* < 0.01) as compared to the vehicle group.

**Figure 7:**
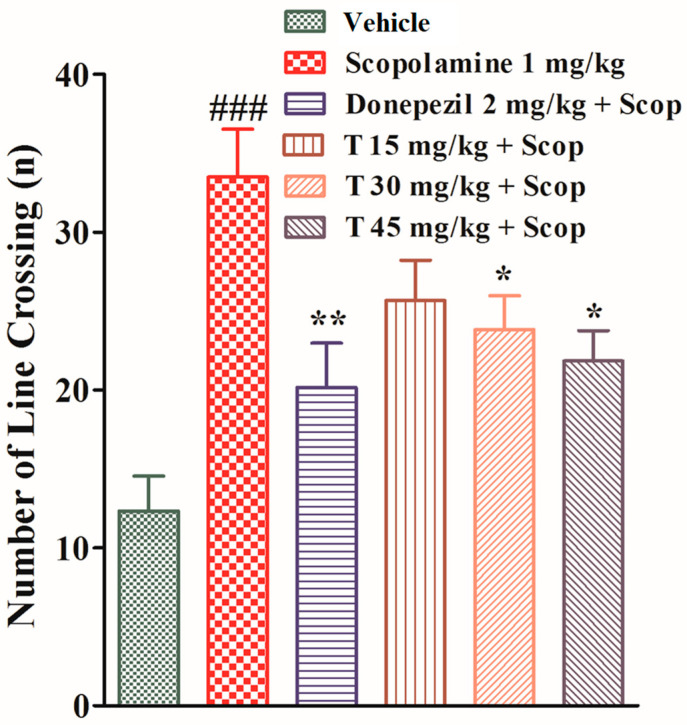
The effect of the test compound on the number of line crossings in the open field test. All values are expressed as mean ± SEM. ^###^ *p* < 0.001 as compared to the vehicle group, * *p* < 0.05 and ** *p* < 0.01 as compared to the scopolamine-only group, one-way ANOVA followed by Dunnett’s post hoc test (*n* = 6).

The donepezil-treated group crossed a significantly smaller number of lines (*p* < 0.01) and showed significantly more rearing (*p* < 0.05) as compared to the scopolamine-treated group. The test-compound-treated group at a dose of 15 mg/kg crossed a lesser number of lines and showed more rearing compared to the scopolamine-treated group but the data were not significant. The test-compound-treated groups at doses of 30 mg/kg and 45 mg/kg displayed significant results (*p* < 0.05) in comparison to the scopolamine-treated group, as shown in Figure 7.

### 2.5. Effect of the Test Compound on the Frontal Cortex and Hippocampal Acetylcholinesterase Enzyme

**Figure 8:**
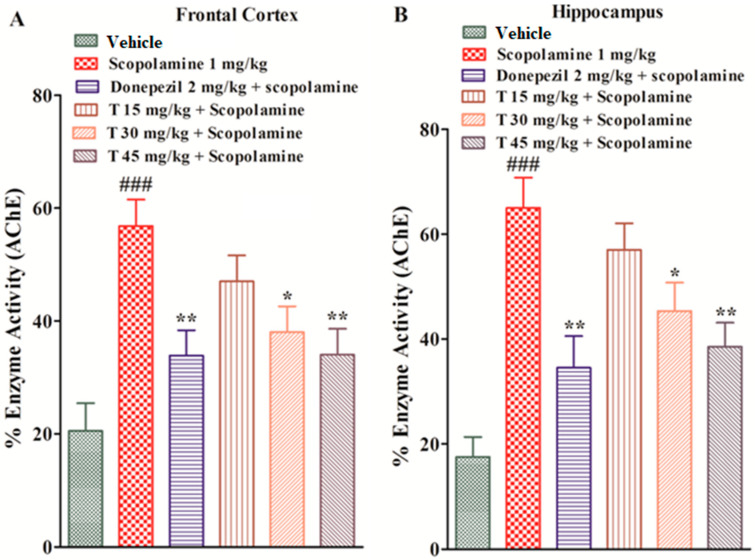
The effect of the test compound on the percent enzyme activity (AChE) in FC (**A**) and HC (**B**). All values are expressed as mean ± SEM. ^###^ *p* < 0.001 as compared to the vehicle group, * *p* < 0.05 and ** *p* < 0.01 as compared to the scopolamine-only group, one-way ANOVA followed by Dunnett’s post hoc test (*n* = 6).

The standard and the test compound groups produced substantial changes in the % enzyme activity of the AChE enzyme in both the frontal cortex and hippocampus. The scopolamine-treated group exhibited significantly (*p* < 0.001) increased AChE enzymatic activity in both the FC and HC, while the test compound groups at various doses displayed a significant decrease in AChE activity (*p* < 0.05, *p* < 0.01), as shown in Figure 8.

### 2.6. Effect of the Test Compound on the Histopathological Examination of the Frontal Cortex and Hippocampus

**Figure 9:**
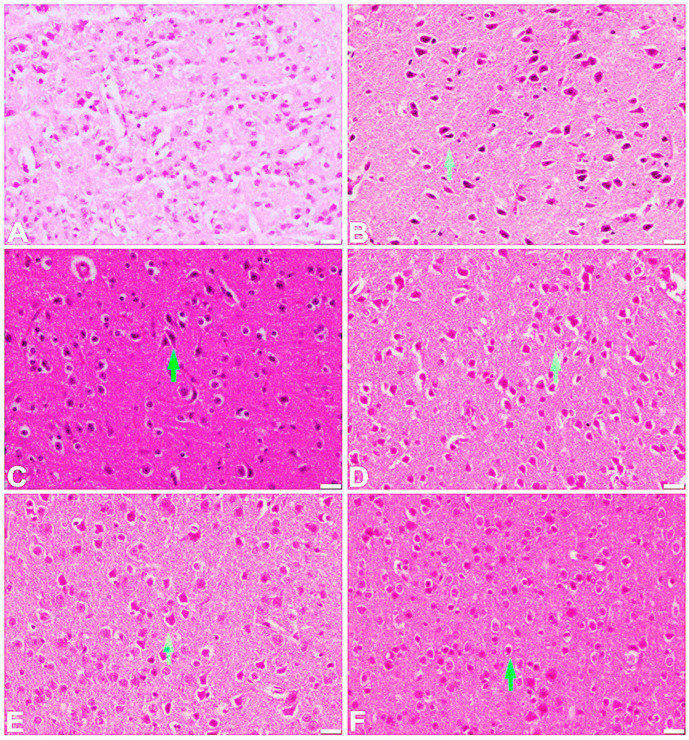
Effect of scopolamine, the test compound at doses of 15, 30, and 45 mg/kg, and donepezil on the frontal cortex (200 µm, H & E). (**A**) Vehicle group: The inner layer of the frontal cortex contains normally appearing pyramidal neurons with adjacent rounded satellite cells. Normal-appearing neutrophils are visible around the pyramidal neurons. (**B**) Scopolamine-treated group: The pyramidal neurons have eosinophilic cytoplasm and shrunken perikarya. (**C**) Test-compound-treated group at a dose of 15 mg/kg: Some regeneration with numerous shrunken pyramidal neurons that show eosinophilic cytoplasm. (**D**) Test-compound-treated group at a dose of 30 mg/kg: Mild to moderate regeneration with shrunken pyramidal neurons. (**E**) Test-compound-treated group at a dose of 45 mg/kg and (**F**) donepezil-treated group show regeneration of pyramidal neurons with normal-appearing neutrophils.

**Figure 10:**
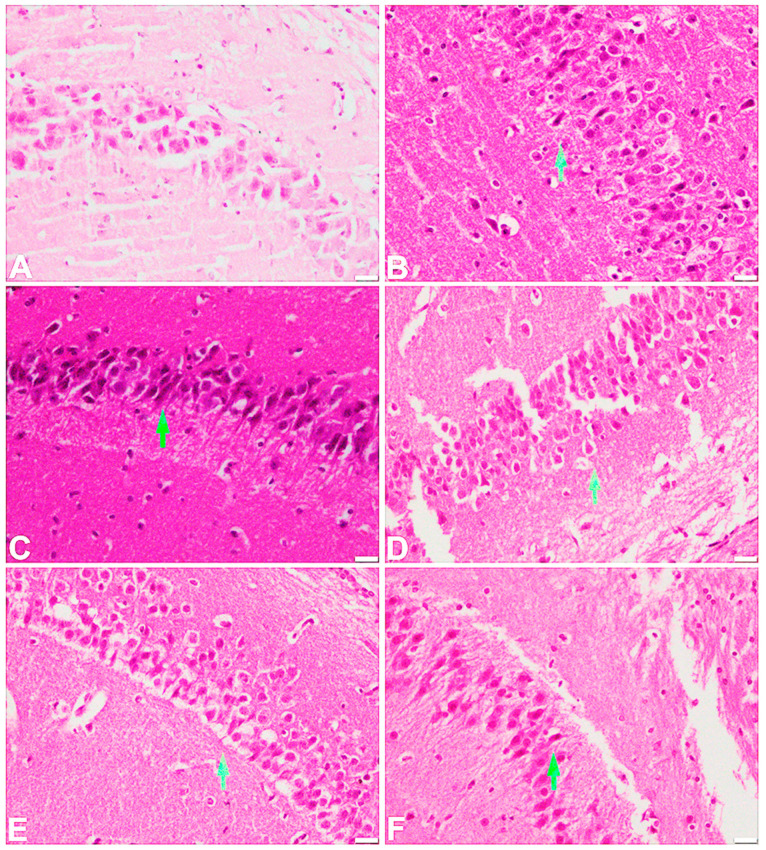
Effect of scopolamine, test compound at doses of 15, 30, and 45 mg/kg, and donepezil on the hippocampus (200 µm, H & E). (**A**) Vehicle group: The large pyramidal neurons appear normal. (**B**) Scopolamine-treated group: There is shrinkage of large pyramidal neurons, the appearance of dark neurons, and glial activation. (**C**) Test-compound-treated group at a dose of 15 mg/kg: Numerous dark neurons with shrinkage of large pyramidal neurons. (**D**) Test-compound-treated group at a dose of 30 mg/kg: Distinct regeneration with mild to moderate shrunken pyramidal neurons. (**E**) Test-compound-treated group at a dose of 45 mg/kg and (**F**) donepezil-treated group are marked by the regeneration of pyramidal neurons with the reduced hyperchromatic appearance of neuronal perikaryon.

The vehicle-treated control group showed a normal appearance of the frontal cortex. The cortical layer consists of large pyramidal neurons with adjacent rounded satellite cells. In the scopolamine-treated group, there was a shrinkage of pyramidal cells with eosinophilic cytoplasm. At the 15 mg/kg dose, there was some regeneration of the shrunken pyramidal neurons, while mild to moderate regeneration was observed at a dose of 30 mg/kg. A marked regeneration of the cortical pyramidal neurons was noted at 45 mg/kg as well as in the standard, donepezil-treated group (Figure 9). A normal histological feature of the large pyramidal neurons of the hippocampal region was observed in the vehicle-treated control group. However, in the scopolamine-administered group, these pyramidal neurons appeared to shrink with an increased distribution of dark neurons and activation of glial cells. The 15 mg/kg group showed the presence of dark neurons with shrinking pyramidal cells. A mild regeneration of the pyramidal cells was observed in the 30 mg/kg treated group, while marked regeneration of these cells along with a reduced hyperchromatic appearance of neuronal perikaryon were noted in the 45 mg/kg treated group as well as in the standard donepezil-treated group (Figure 10). Table 2 shows the results of our histological examination of all groups in the frontal cortex and hippocampus.

## 3. Discussion

Alzheimer’s disease is the most frequent, neurodegenerative, neurological syndrome, marked by slow memory loss, behavioral changes, and cognitive dysfunction [1,2]. The principal pathophysiological causes of AD include neuronal damage due to oxidative stress, production and aggregation of abnormal beta-amyloid proteins, tau protein phosphorylation, insufficient production of acetylcholine, inflammation in the brain, and changes in calcium metabolism [5]. In the field of neurosciences, AD treatment is the most unmet medical need, and current medications relieve the symptoms but do not have a significant impact on disease progression [11].

Animal models of AD induced by chemicals can help researchers better understand the illness and therapy options. For induction purposes, different chemicals are used such as scopolamine, streptozotocin, okadaic acid, and colchicine [12]. In the current study, scopolamine, a competitive cholinergic antagonist, was used to induce cholinergic dysfunction, increased AchE activity in the frontal cortex and hippocampus, and produce Alzheimer-like dementia in the experimental animals.

Currently, there are only two strategic treatments available that are clinically approved for the treatment of Alzheimer’s disease, i.e., acetylcholine esterase inhibitors, which include donepezil, tacrine, galantamine, rivastigmine, and N-methyl-D-aspartate receptor antagonist (memantine), but these are only used for symptom improvement and have poor bioavailability, non-selectivity, limited therapeutic effect, a narrow therapeutic window, hepatotoxicity, and adverse peripheral cholinergic side effects, which limit the use of these drugs. Tetrahydro-2H-1,3,5-thiadiazine-2-thione derivatives are of remarkable importance. Keeping in mind the importance of tetrahydro-2H-1,3,5-thiadiazine-2-thione derivatives, this study was designed to evaluate the ameliorative potential of THTT against scopolamine-induced AD. Primarily, the test compound was subjected to an acute toxicity test, and the LD50 of the test compound was evaluated and was proven to be well-tolerated, because no behavioral change or mortality was observed up to a dose of 500 mg/Kg, and the compound showed a good safety profile. Antioxidant DPPH free radical scavenging and AChE enzyme inhibition assays were performed.

Neurodegenerative disorders result in the formation of ROS in the brain cells, as well as the release of cytochrome C, a cascade of caspases (a group of proteases that mediate apoptosis), and DNA damage. Scopolamine-induced impaired memory has been linked to increased oxidative stress, increased Ca^2+^ influx, and neuronal death [13]. The antioxidant activity of the test compound, as determined by using an antioxidant DPPH free radical scavenging assay, exhibited an inhibitory concentration of 75 μg/mL at 1000 μg/mL concentration with an IC50 value of 97.75 μg/mL, which reflects that compound’s antioxidant property.

Acetylcholine deficiency is thought to be the most common cause of Alzheimer’s disease, and acetylcholinesterase inhibitors are most frequently used for the symptomatic relief of Alzheimer’s disease. Suggested mechanisms include blocking the AChE enzyme, preventing cytokines’ release from the monocytes and microglia, and preventing cells from free radical toxicity and damage caused by beta amyloids [14]. The anti-cholinesterase potential of the test compound exhibited 73.84 μg/mL of AChE inhibition at a 1000 μg/mL concentration with an IC50 value of 69.41. The results were compared with a donepezil-instigated IC50 value of 8.65 μg/mL. This study showed that our selected test compound exhibited an anti-cholinesterase property that is helpful in AD management. AChE remains a high-potential target in the symptomatic relief of AD because cholinergic deficiency is a persistent and early finding in Alzheimer’s disease. To further elucidate the cholinergic hypothesis, we examined whether the test compound affected acetylcholinesterase enzymes in the frontal cortex and hippocampus following its administration. Our study suggests that the selected test compound exhibits anti-cholinergic properties by inhibiting the AChE enzyme in both the hippocampus and frontal cortex, as we found in the experimental animals.

The substantial ameliorative effect of the compound was instigated by reducing the escape latency and improving the total time spent by the experimental animals in the target quadrant in the MWM test. In the EPM experiment, the test compound showed a dose-dependent decrease in the transfer latencies, i.e., ITL and RTL, which reflects an improvement in learning and memory behavior as mice avoid open spaces and heights. An open field experiment is widely used to examine anxiety, locomotion, and other activities that consist of exploratory behavior in experimental animals [15]. Our selected test compound significantly reduced the number of line crossings and significantly increased the rearing as compared to the scopolamine-treated group. This shows an improvement in the locomotor effect as scopolamine increases the ambulation (number of line crossings) and decreases the open rearing [16].

The hippocampus and frontal cortex play an important role in learning and memory. That is why these two areas are important from the perspective of AD. In this study, H and E staining was performed to examine the different pathological changes instigated by scopolamine in the brains of the experimental mice. The test compound significantly reduced the pathological changes such as pyknosis, karyolysis, vacuolation, and fibrosis induced with the help of scopolamine in the mice’s brains in both the hippocampus and frontal cortex. The results for the test compound were almost comparable with those for the standard drug donepezil. As a result, the findings of our investigation suggest that the chosen test compound has disease-modifying and neuroprotective qualities, and so it could be effective in the treatment of AD.

## 4. Materials and Methods

### 4.1. Chemicals and Reagents

3,5-*bis*(2-hydroxyethyl)-1,3,5-thiadiazinane-2-thione was obtained from the laboratory of Dr. Rasool Khan, Associate Professor at the Institute of Chemical Sciences. We also sourced scopolamine (Sigma, St. Louis, MO, USA), donepezil (Sigma, St. Louis, MO, USA), acetylcholinesterase enzyme (Sigma-Aldrich, MO, USA), acetylthiocholine iodide (Sigma-Aldrich, MO, USA), absolute ethanol (Merck, Darmstadt, Germany), ascorbic acid (Sigma-Aldrich, USA), 2,2-diphenyl-1-picrylhydrazyl (DPPH) (Sigma-Aldrich, MO, USA), DTNB (Promega, Madison, WI, USA), ethanol, dimethyl sulfoxide (Sigma-Aldrich, MO, USA), formaldehyde, potassium hydroxide, methanol, magnesium chloride, normal saline, paraffin wax, sucrose (Sigma Aldrich, MO, USA), and xylene (RCI Lab Scan, Shannon, Ireland). Tween 80, hematoxylin, and eosin were procured from Scharlau Chemicals, Spain.

### 4.2. Biochemical Assays

#### 4.2.1. Acetylcholinesterase (AChE) Inhibition Assay

The AChE inhibition method was based on the hydrolytic breakdown of acetylthiocholine iodide by an AChE enzyme, which results in the formation of the 5-thio-2-nitrobenzoate anion. This anion forms a complex with DTNB, which results in the formation of a yellow-colored product, as measured using a UV–visible spectrophotometer. Donepezil was used as a standard for comparison with the test compound. Following Elman’s assay, both the standard and test drug solutions were made in various concentrations ranging from 62.25 μg/mL to 1000 μg/mL. KOH solution was used to adjust the pH of the solutions. Enzymatic solutions for the assay were made by dissolving AChE (518 U/mg) in phosphate buffer (pH 8.0). The solutions were further diluted to a final concentration of 0.03 U/mL. Distilled water was used to make the DTNB (0.0002273 M) and acetylthiocholine iodide solutions. The DTNB and test samples were added after the addition of 5 mL of enzyme solution. The mixture was then heated to 30 °C for 15 min before adding the substrate acetylthiocholine iodide (5 μL). The absorbance was measured at 412 nm by using a UV–visible spectrophotometer. The absorbance was recorded with the reaction time, and experiments were performed in triplicate. Finally, the absorbance rate with the change in time was used to determine enzyme activity and enzyme inhibition by standard and test compounds.

% enzyme activity = (V/V max) × 100;

% enzyme inhibition = (100% activity of enzyme);

V max = rate of reaction without inhibitor;

V = rate of reaction in inhibitor’s presence.

#### 4.2.2. 1,1-diphenyl, 2-picrylhydrazal Free Radical Scavenging Assay

The antioxidant activity of the test compound THTT was analyzed by using a 1,1-diphenyl, 2 picrylhydrazal (DPPH) free radical scavenging assay, and the result was detected as a change in color from deep violet to light yellow [17,18,19]. The DPPH solution was prepared in 100 mL methanol. A stock solution (1 mg/mL) of the test compound was also prepared and diluted to concentrations ranging from 62.25 μg/mL to 1000 μg/mL. Equal concentrations of the test drug and DPPH was mixed and incubated at 23 °C. After incubation, the absorbance was measured at 517 nm using a UV–visible spectrophotometer. Ascorbic acid was used as a standard with the same solution and concentrations as the test compound. The test compound dissolved in methanol was used as a blank. The result was calculated by the formula

% DPPH activity = (X − X1) × 100

where X = absorbance of the control, and X1 = absorbance of the test compound.

### 4.3. In Vivo Study

#### 4.3.1. Animals

BALB/c mice were used in this study after breeding in the animal house facility. The animals were kept at 22 ± 2 °C under a light–dark cycle of 12/12 h with food and water provided ad libitum. The experimental procedures were approved by the Ethical Committee of the Department of Pharmacy, University of Peshawar (UOP) with reference number 02/EC-18/Phar, dated 16 October 2018. This study was performed following the rules and regulations of the UK Animals (Scientific Procedures) Act 1986.

#### 4.3.2. Drug Solubility and Administration Protocol

In this study, THTT was dissolved in a freshly prepared vehicle consisting of normal saline, DMSO, and Tween 80 in a ratio of 97:2:1, respectively. All the solutions were prepared daily before drug administration. The drug was administered intraperitoneally once daily for 33 days to all groups, except the scopolamine-treated group. Starting from the 23rd day, scopolamine was administered intraperitoneally to all groups, except the vehicle group, 30 min after the standard and test compound administration, continuously for 11 days. On the 33rd day, brain samples were collected for biochemical and histological studies.

#### 4.3.3. Acute Toxicity Test

For the evaluation of the safety profile, an acute toxicity test was performed using mice of either sex. The test compound was administered at 15, 30, 60, 120, 240, and 500 mg/kg doses, respectively. The animals were observed initially for 2 h, and then after 24 h, for any unusual behavior such as writhing, ataxia, righting reflex, aggressiveness, and convulsions [20,21].

#### 4.3.4. Animal Grouping

The experimental animals were separated into six groups, each consisting of six mice:

Group 1 (vehicle control) = This group received saline: DMSO: Tween 80 in the ratio of 97:2:1;

Group 2 (scopolamine group) = This group received only scopolamine at 1 mg/kg;

Group 3 (donepezil group) = This group received donepezil at 2 mg/kg + scopolamine at 1 mg/kg;

Group 4 (test compound 15 mg/kg) = This group received the test compound at 15 mg/kg + scopolamine at 1 mg/kg;

Group 5 (test compound 30 mg/kg) = This group received the test compound at 30 mg/kg + scopolamine at 1 mg/kg;

Group 6 (test compound 45 mg/kg) = This group received the test compound at 45 mg/kg + scopolamine at 1 mg/kg.

#### 4.3.5. Elevated Plus Maze (EPM)

The EPM is a behavioral test that is used to check memory and learning. It comprises two open arms (30 cm length, 6 cm width) and two enclosed arms of the same size, opposite to each other, with 15 cm high walls and 40 cm elevation from the ground, taking the appearance of a plus sign. During the trial, the experimental animal was placed at the end of an open arm, facing it away from the center, and allowed to move. The mouse was allowed to explore the EPM for 90 s and the latency time was recorded. The same trial was performed after 24 h of scopolamine administration to determine retention transfer latency (RTL) [22].

#### 4.3.6. Y-Maze Test

The Y-maze, an exteroceptive behavioral test for short-term memory, has three arms, fixed at equal angles (120° angles), and each arm is 30 cm long, 8 cm wide, and 20 cm high. The experimental animal was placed in one of the arms and allowed to explore the maze for five minutes after the administration of scopolamine [23]. The results were calculated by the formula

% spontaneous alteration = (actual alteration/total number of arm entries) − 2 × 100

#### 4.3.7. Light and Dark Box Test

This test is designed to analyze the learning skills in BALB/c mice and is based on the idea that all rodents prefer a dark chamber over a light one. On the first day, after 30 min of scopolamine administration, the experimental animal was placed in the light compartment and the time spent in the light and dark compartments was examined for 5 min, respectively. On the second day, a test trial was performed after 24 h of scopolamine administration [4].

#### 4.3.8. Morris Water Maze Test

The Morris water maze (MWM) assesses spatial learning and recognition behavior using a circular pool divided into four quadrants. In the training phase, transparent water and visible boundaries aid mice in locating a 10 cm diameter platform slightly above the water’s surface. In the test trial, the water becomes opaque, and the platform is positioned 1 cm below the surface. The test trial consisted of five days and started after 30 min of the administration of scopolamine. Mice from each group were subjected to two test trials per day and were allowed to find the platform within 90 s, and then allowed to sit on the platform for about 30 s to recognize the platform location. The escape latency of each animal was recorded carefully. A probe trial was performed after 24 h of scopolamine administration on day six. The experimental animals were allowed to explore the maze for 60 s. The time spent by each animal in the target quadrant was recorded with the help of a camera [24,25].

#### 4.3.9. Open Field Test

The OF test is used to evaluate the locomotion (number of line crossings) and rearing of mice [26]. The apparatus consists of a wooden box divided into four equal parts (45 × 45 × 40 cm). By painting lines (4 vertical and 4 horizontal) on the base of the open field apparatus, sixteen squares of 20 × 20 cm are created. The open field consists of a single trail. Each animal was placed in the middle of the apparatus and allowed to explore the open field for five minutes. Each rearing and line crossing was noted during this period. A camera was used to record the responses of the animals [27,28].

#### 4.3.10. Assessment of Acetylcholine Esterase in the Frontal Cortex and Hippocampus

The frontal cortex (FC) and hippocampus (HC) were taken out and placed in ice-cold 0.1 molar phosphate-buffered saline (PBS) (pH 8). After weighing, 20 mg tissue/mL homogenates were prepared for each brain sample by homogenizing the dissected regions in PBS (pH 8.0). Homogenization was carried out using a tissue grinder homogenizer (Daigger Scientific Inc., 620 Lakeview Parkway, Vernon Hills, IL, USA, 6006). The resulting homogenates underwent centrifugation at 10,000 rpm for 10 min at 4 °C, followed by centrifugation at 1000× *g* at 4 °C for 10 min and evaluation of the supernatant liquid for the AchE assay. The supernatant was added to the cuvette, containing DTNB (10 mM), PBS (0.1 M, pH 8.0), and 50 μL of each test solution, followed by acetylthiocholine (75 mM) introduction as a substrate, and then the final solution was incubated for 3 min and 37 °C.

#### 4.3.11. Histopathological Evaluation

Brain samples were analyzed for different histopathological changes in the brain, such as pyknosis, karyolysis, fibrosis, and vacuolation. The isolated brain already washed with ice-cold phosphate-buffered saline (PBS) was allowed to soak in 10% paraformaldehyde solution for 10 h, and then the specimen was shifted to 30% sucrose solution in 0.1 mol/L PBS [24]. All the tissues were dehydrated in an automatic tissue processor and finally subjected to molten paraffin. The brain specimens were cut and attached to the slides. All the tissues were then dewaxed and rehydrated followed by staining with hematoxylin and eosin.

#### 4.3.12. Statistical Analysis

All the results were expressed as mean ± standard error of the mean (SEM). Data were analyzed by one-way analysis of variance (ANOVA) followed by a post hoc Dunnett’s test. The statistical analyses were conducted using GraphPad Prism 5 (GraphPad Software Inc., San Diego, CA, USA). A value of *p* < 0.05 was considered significant.

## Figures and Tables

**Table 1:** Shows the effect of the test compound in the DPPH free radical scavenging assay.

Sample	Concentration (μg/mL)	%-DPPH Free Radical Scavenging Activity (Mean ± SEM)	IC_50_ (μg/mL)
Test Compound	1000	75 ± 3.0	97.75
	500	65 ± 2.7	
	250	50 ± 2.9	
	125	41.7 ± 4.2	
	62.5	28.3 ± 4.41	
Ascorbic Acid	1000	80 ± 3.5	67.98
	500	71 ± 3.8	
	250	60 ± 3	
	125	51 ± 5	
	62.5	28.3 ± 4.4	

**Table 2:** Histological examination of all groups (frontal cortex + hippocampus).

Frontal Cortex Region					
S. No.	Group	Degeneration Process			Plaques/Fibrosis
		Pyknosis	Karyolysis	Vacuolation	
1	Vehicle group	No	No	No	No
2	Scopolamine	Yes	Yes	Yes	Yes
3	Scopolamine + donepezil	No	No	Yes	No
4	T15 + scopolamine	Yes	Yes	Yes	No
5	T30 + scopolamine	No	No	Yes	Yes
6	T45 + scopolamine	No	No	Yes	No

## Data Availability

The datasets generated during and/or analyzed during the current study are available from the corresponding author on reasonable request.
